# Comparative analysis of explainable machine learning prediction models for hospital mortality

**DOI:** 10.1186/s12874-022-01540-w

**Published:** 2022-02-27

**Authors:** Eline Stenwig, Giampiero Salvi, Pierluigi Salvo Rossi, Nils Kristian Skjærvold

**Affiliations:** 1 Department of Circulation and Medical Imaging, The Norwegian University of Science and Technology, Trondheim, Norway; 2 Department of Electronic Systems, The Norwegian University of Science and Technology, Trondheim, Norway; 3 Clinic of Anaesthesia and Intensive Care Medicine, St. Olav’s University Hospital, Trondheim, Norway

**Keywords:** Machine learning, Explainability, Mortality prediction, eICU, SHAP values

## Abstract

**Background:**

Machine learning (ML) holds the promise of becoming an essential tool for utilising the increasing amount of clinical data available for analysis and clinical decision support. However, the lack of trust in the models has limited the acceptance of this technology in healthcare. This mistrust is often credited to the shortage of model explainability and interpretability, where the relationship between the input and output of the models is unclear. Improving trust requires the development of more transparent ML methods.

**Methods:**

In this paper, we use the publicly available eICU database to construct a number of ML models before examining their internal behaviour with SHapley Additive exPlanations (SHAP) values. Our four models predicted hospital mortality in ICU patients using a selection of the same features used to calculate the APACHE IV score and were based on random forest, logistic regression, naive Bayes, and adaptive boosting algorithms.

**Results:**

The results showed the models had similar discriminative abilities and mostly agreed on feature importance while calibration and impact of individual features differed considerably and did in multiple cases not correspond to common medical theory.

**Conclusions:**

We already know that ML models treat data differently depending on the underlying algorithm. Our comparative analysis visualises implications of these differences and their importance in a healthcare setting. SHAP value analysis is a promising method for incorporating explainability in model development and usage and might yield better and more trustworthy ML models in the future.

## Background

With the increasing availability and use of digital aid in health care, such as sensors and electronic health records, patients generate large amounts of data that can be used in treatment and analysis. Some of this information is not necessarily informative on its own but can give insight into complex medical problems when combined. Statistical modelling has long been one of the main approaches in medical research when studying relationships and their significance regarding different variables. However, due to data availability and better hardware, the use of artificial intelligence (AI) and machine learning (ML) has increased rapidly within this field over the last few years, supplementing, and to a certain degree replacing, the traditional statistical models [1].

Statistical modelling and ML can both be used for inference and prediction but have somewhat different approaches. Traditional statistical modelling should utilise pre-analytical clinical assumptions regarding the underlying structure of the data, while ML models often are purely ‘data-driven’ and are developed by generalising patterns within certain constraints specific to the algorithms [2].

Predictions in standard statistical models are often ‘human-readable’ to a certain degree. The opposite is the case with ML models, which are often compared to ‘black boxes’ where the mapping between the input feature and prediction is not clear. Not only for the end-user, but also the developer. The importance of different features on a prediction can for some ML models be explained using coefficients, or by tracing or visualising the steps taken by the algorithm. Still, this is not always sufficient for obtaining a model that is easily understood by humans for development and use. The complexity of a model increases rapidly with an increasing number of features, and explaining the impact of individual features is not straightforward. Without understanding, the models cannot be trusted to perform according to our expectations. There is an implementation gap for ML in healthcare, where a lack of trust in the model plays a vital part [3]. Models that are not trusted will not be used. The need for explainable ML and models that can be easily understood by humans is becoming increasingly apparent [4, 5].

A recently developed tool for making ML models more intuitive is SHapley Additive exPlanations (SHAP) [6] which are based on Shapley values [7]. Shapley values are a solution concept from game theory that weighs the contribution of each player and distributes the ‘payout’ accordingly. An implementation of this game theory provides weights or relations describing how big a role different features play in determining the output of the model. Some studies published over the last few years have incorporated SHAP or similar tools as part of the model development and performance evaluation [8–10]. This applies only to a small portion of published studies, and there is yet work to be done before explainability become state-of-the-art.

ML models need to be evaluated thoroughly to find their true performance regarding the intended purpose. Many ML models are usually evaluated using only a few performance metrics. This, in combination with the lack of transparency, often leads to poor evaluation of certain aspects of the model and model performance. A recent systematic review of studies where ML is used for predicting mortality based on ICU data [11] showed that papers generally only focus on the discriminative capabilities of models. Additionally, the papers rarely reported metrics related to other evaluation methods, such as calibration, i.e. how well the distribution of predicted probabilities matches the expected distribution. Models should be evaluated based on the use-case they were developed for, and the use of solely one metric would be highly insufficient in most cases [12, 13]. Domain knowledge is an integral part of ML model explainability and trustworthiness, and should be applied in all stages of the model development and implementation. This is particularly important in healthcare, where models should reflect the human physiology. The models should be correct for the right reasons, and medical experts are an essential part of this.

There are many potential uses for ML in healthcare, with tasks ranging from cancer detection [14] to predicting hospital readmission [15] or mortality [16]. Mortality prediction for patients in the Intensive Care Unit (ICU) can be regarded as a simple classification problem with two possible outcomes: dead or alive at ICU, or hospital, discharge. The model utilises variables such as *age*, *height* and *weight*, vital values, lab values, and diagnoses, with features ranging from highly granulated temporal data to single values such as the mean of the first 6 hours of the hospital stay or discrete variables like *age* or sex. There are many potential uses of mortality predictions, both on individual and group level, including stratifying and identifying patients, comparing and improving ICUs, helping with clinical decision making, knowledge derivation, and resource allocation [17, 18].

With the advent of personalised medicine, predictions on the individual patient level are warranted. However, models developed for groups are not directly applicable to individuals, as the mortality prediction reflects the probability of survival or death in a group or cohort [19]. Similarly, it is not possible to take a model developed for a specific patient group and use it in a different group [19]. Hence, the intended use of the model is crucial.

In this study, we want to investigate how individual features impact predictions from different ML models to learn how they compare to common medical theory, and to each other. This is done by developing four hospital mortality prediction models from a publicly available dataset using the same input features to highlight similarities and differences between the models from an end-user point of view using SHAP-values. Dataset-level performance metrics are calculated for the different ML models to assess the overall performance and compare it to the well-known APACHE IV score as a baseline. The purpose of the study is not to find the best model regarding explainability, but to clarify aspects to be aware of when developing and using ML models, and to explore why the ability to explain ML models, and not just the model output, is needed.

## Methods

This section is divided in three parts: *Input*, *Model* and *Output*, representing the principal components of an ML model. The analysis is done with Python, and the code is available online.

### Input

#### The dataset

The dataset used in this study is the freely available multi-centre eICU Collaborative Research Database [20], which contains information about patients admitted to critical care units in the US between 2014 and 2015. The dataset includes over 200 000 ICU stays from more than 139 000 patients and holds information such as patient demographics, lab values, information about diseases and treatment, and vital values with a resolution of 5 minutes. A large part of the dataset is dedicated to the Acute Physiology and Chronic Health Evaluation (APACHE) IV severity-of-disease classification system [21], and the dataset includes designated tables for the parameters used to calculate this score.

#### Patient selection

**Fig. 1:**
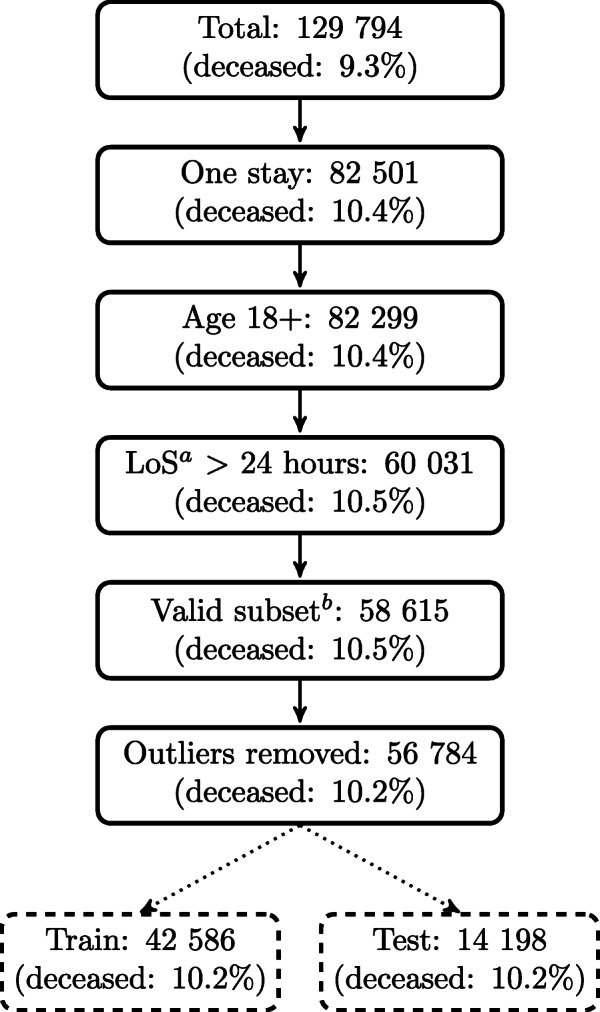
Patient selection. Patient selection criteria and train/test splitting of dataset. ^*a*^Length of stay. ^*b*^Patients with valid features: *age*, sex, patient id, discharge status, *BMI*, predicted hospital mortality, admission diagnosis

The patients’ inclusion criteria are shown in Fig. 1, which also includes the training and test set selection described later. Patients with multiple ICU stays are excluded, as well as patients younger than 18 years. Patients with a stay of fewer than 24 hours are excluded to weed out patients that are in the ICU for a short stay before death or transferral. Patients without valid *age*, sex, patient id, discharge status, body mass index (*BMI*), admission diagnosis, or predicted hospital mortality (based on APACHE IV) are also excluded. Outliers for *height* and *weight* are removed manually, while values outside of five standard deviations are removed for vital and lab values.

#### Feature selection

The selected features are the same as for calculating the APACHE IV score, with some exceptions. *Height* and *weight* are combined in one variable: *BMI*. Glasgow Coma Scale (*GCS*) is used as a combined score for *eyes*, *motor* and *verbal*. The features *PaO2* and *FiO2* are combined into one feature: *pfratio*.

Feature extraction is simplified using the tables containing the features used for calculating the APACHE IV score.

The vital and lab values used are the ‘worst’ value for each feature, i.e. the value furthest away from a reference value in the first 24 hours in the ICU. Many of the patients have stayed in the hospital prior to the ICU, and treatment will also affect the values. The result will reflect treatment and care given during the entire stay, and events can occur after the first 24 hours that are not considered by the models.

#### Train/test set

Most ML models are developed with the help of a training set and then validated on a test set to evaluate how the model performs on previously unseen data. The train/test split is also shown in Fig. 1. The training set comprises 75% of the patients, and the test set comprises the remaining 25%. The share of deceased patients is the same in both sets (10.2%). The same training set and test set are used for all models.

The ML methods considered cannot handle missing inputs. The missing numerical values are therefore filled with the mean of the training set. Patient with missing categorical variables are not included in the study.

### Models

We construct four different ML models; Random Forest (RF), Logistic Regression (LR), Adaptive Boost Classifier (ADA), and Naive Bayes (NB). All models are from the scikit-learn Python package [22]. RF and ADA are both tree ensemble models. The models comprise multiple decision trees that when combined give better results than individual trees. Decision trees determine the output by using a flowchart-like structure to impose a series of conditions on the input. The RF model comprises multiple trees trained in parallel on different subsets of data before the final result is found by majority vote. The ADA model uses the same dataset for each tree, but the trees are trained sequentially instead of in parallel with trees updated based on the previous tree’s mistakes. The result is decided by a weighted majority vote. LR models resemble linear regression, but the output variable is binary. The NB classifier is based on Bayes Theorem and assumes conditional independence between input features.

Several different pre-processing techniques are tested to see if they affect the results significantly. This includes scaling of the input features, removal of patients with more than X number of missing values, and filling the missing values with (APACHE IV) reference values instead of mean values. Different ratios between deceased and alive patients in the training set are also tested. The models are trained by minimising the error with respect to the area under the receiver operating characteristic curve (AUC ROC/AUC/c-statistic).

### Output

The hospital mortality prediction can be presented as a probability, or solely as a binary outcome based on a risk threshold or operation point. Probabilities facilitate risk stratification of patients and allow a more nuanced understanding than simple ‘alive’/‘deceased’ predictions. However, a probability still lacks information useful for clinical decision support.

The AUC is a popular metric for evaluating a model’s discriminative abilities, i.e. how well the model separates the classes. A perfect classifier will have an AUC of 1, while a random classifier yields an AUC of 0.5. AUCs for different models tested on the same dataset are often directly compared to determine which one performs better in terms of discrimination.

The AUC confidence intervals are found by bootstrapping with 10 000 bootstrap samples, each of the size of 70% of the test set.

While AUC is used to evaluate the models’ discriminative abilities, calibration curves are plotted to evaluate the calibration. Calibration is the agreement between the observed and predicted risk [23] and can be visualised with calibration curves where the predicted probability (x-axis) is plotted against the observed frequency (y-axis). A model capturing the accurate risk estimation would have the calibration curve *y*=*x*.

#### SHAP

The method for finding SHAP values differs depending on the type of model. It is possible to find exact SHAP values for tree models and linear models, while estimations are found for other types of models using a weighted local linear regression. This model agnostic method for finding SHAP values does not make any assumption about the model and is, therefore, slower than other methods. Because of the time and resources needed for this model agnostic method are SHAP values often based on a small subset of the data. The NB model is the only model that requires the use of this model agnostic method. We used 1000 samples for the evaluation in this case. For calculating the SHAP values for the LR model, a correlation between the features is assumed.

The SHAP values cannot be compared directly between models due to scaling differences. Still, it is possible to compare how different models weigh different input features by considering the shape of the different plots.

## Results

### Area under the receiver operating curve

**Fig. 2:**
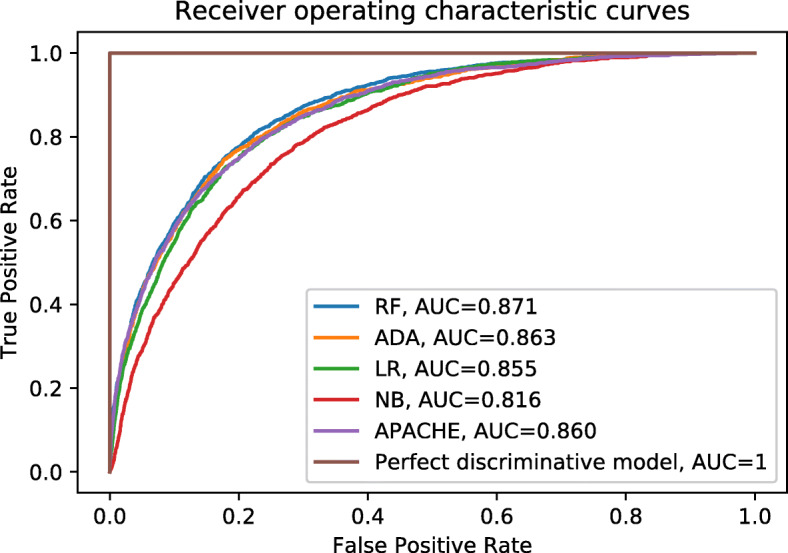
Receiver operating characteristics (ROC) curves. The true positive rate is plotted against the false positive rate for different risk thresholds. A perfect classifier has an AUC of 1, while a random classifier will have an ROC curve along the line *x*=*y* and an AUC of 0.5

The receiver operating characteristic curves are plotted in Fig. 2, and the AUCs are listed in Table 1. The RF model has the highest AUC, followed by the ADA model, APACHE IV, LR and NB. The ADA and APACHE IV models have almost completely overlapping confidence intervals. These two confidence intervals are also partly overlapping with both RF and LR. The NB model is the only model without any overlapping confidence intervals.

**Table 1:** Area under the receiver operating characteristics curves

Model	AUC [CI^a^]
RF	0.871 [0.865, 0.877]
ADA	0.863 [0.857, 0.869]
LR	0.855 [0.849, 0.862]
NB	0.816 [0.810, 0.823]
APACHE	0.860 [0.854, 0.867]

^a^Confidence interval

### Calibration curves

**Fig. 3:**
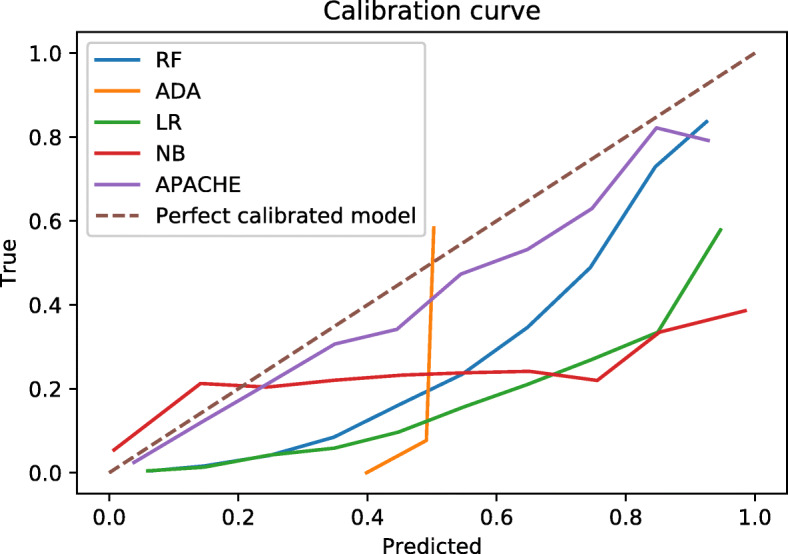
Calibration curves. Illustrate how well the predicted probabilities match the actual mortality

The calibration curves are shown in Fig. 3. All calibration curves lie below the line *y*=*x* apart from a small part of the NB model calibration curve, i.e., the predicted mortality is higher than the actual mortality. Inspecting the curve for the RF model, the mortality for the group of patients with a predicted mortality of 60% is in reality 30%. This means that the chances of survival are better than what the model predicts.

### SHAP summary plot

**Fig. 4:**
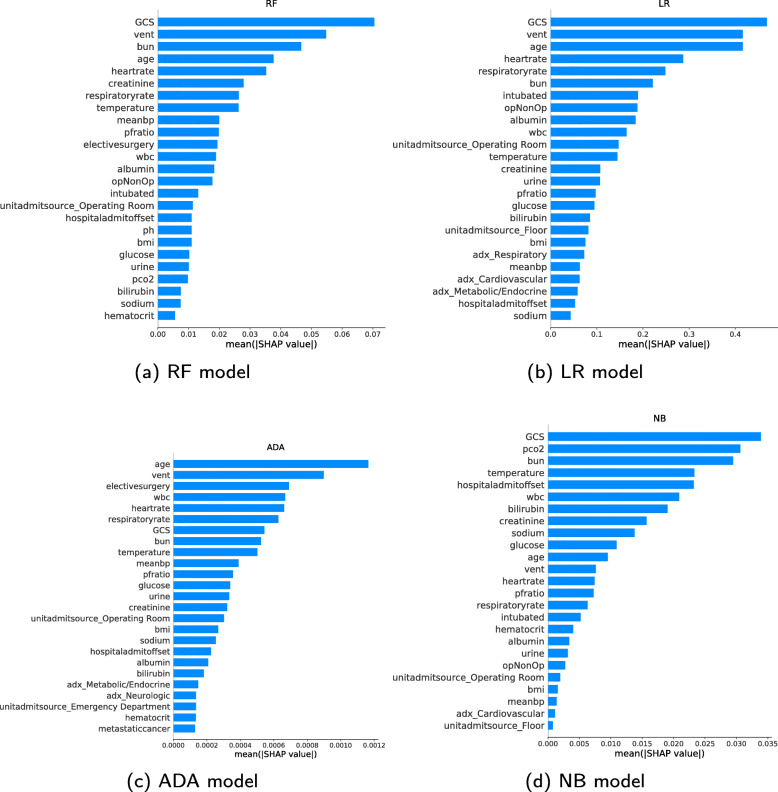
SHAP summary bar plots. Feature importance for the different ML models listed top-down. The longer the bar, the larger impact the feature has on the output

Figure 4 shows the feature importance for the models with respect to the mortality-prediction task. The features are listed top-down with decreasing importance. Only the top 25 features are listed, and categorical variables are split into one bar per category. The sum of the contribution from each category gives the total contribution before the one hot encoding. The bar lengths show the average impact of the individual features on the models’ output. For the RF model, the *GCS* is the most important feature followed by *vent*^1^ and blood urea nitrogen (*bun*). All models apart from ADA place *GCS* as the most important feature. *Age* is also listed as an important feature by all the models, as well as *vent* and *GCS*.

**Fig. 5:**
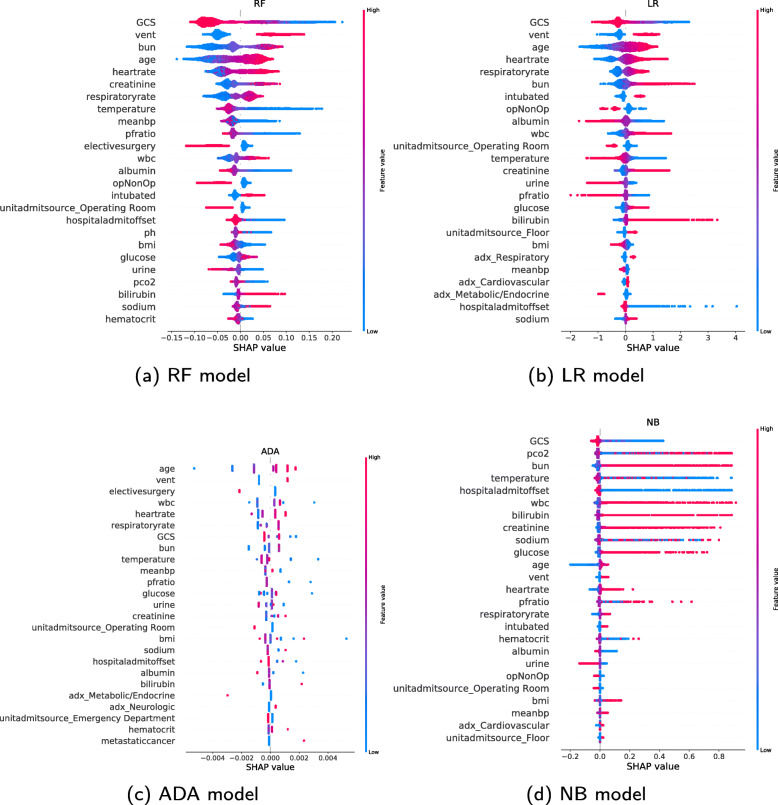
SHAP summary plots. Feature importance for the different ML models listed top-down. Each dot represents the impact on the prediction from a specific patient’s feature value. Dots to the left of the line *x*=0 are considered by the model to decrease the chances of mortality, while the dots to the right are considered to increase the chances

A different presentation of the SHAP summary plots can be seen in Fig. 5. The order of the features listed is the same as in Fig. 4, and the x-axis shows the SHAP values for individual patients instead of the average absolute value. The further away from the vertical line at *x*=0, the larger the impact on the output prediction. Values to the left are contributing to increased chances of survival, while values to the right are pushing the prediction towards increased mortality. The colourful vertical lines are made of dots, with one dot for each patient. The colour of a dot signifies the feature value for that patient. A pink dot represents a high value, while a blue dot represents a low value. The gradients represent the values in between. These plots visualise how different feature values contribute to either survival or death, but the total contribution from each feature is less prominent compared to the bar plots.

### SHAP force plot

**Fig. 6:**
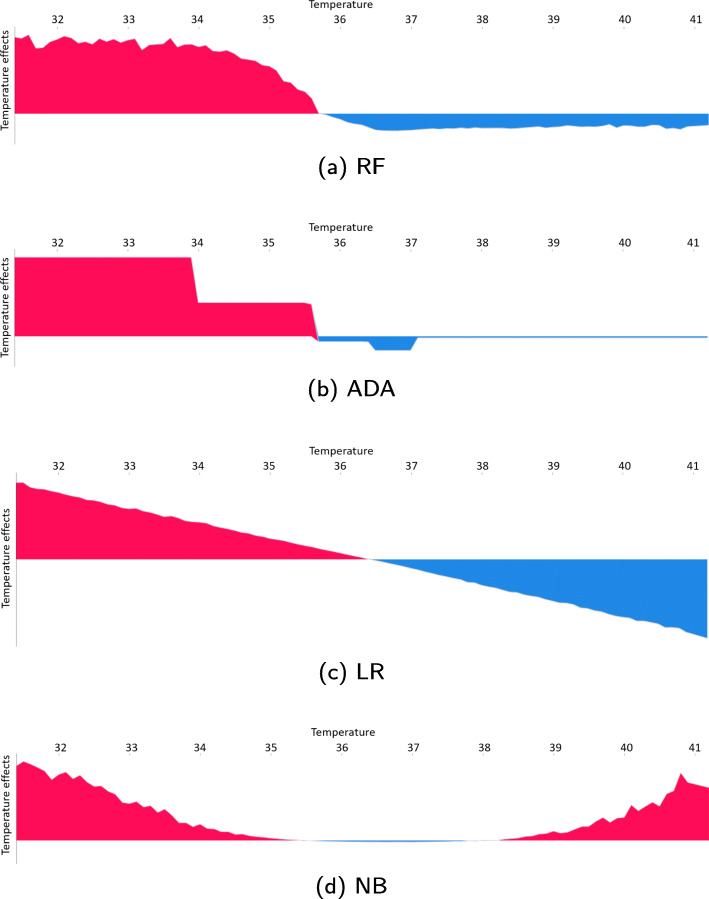
SHAP force plot for temperature. The y-axis represents the average effect of the temperature values listed along the x-axis. The pink sections are where the temperature pushes the prediction towards mortality while the blue sections are where the temperature pushes the prediction toward survival

**Fig. 7:**
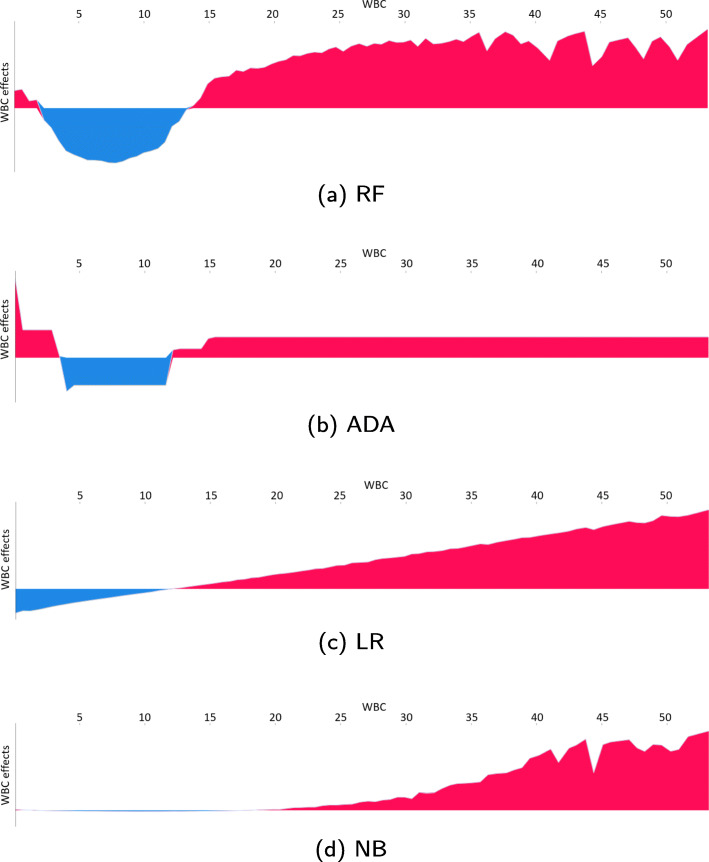
SHAP force plot for white blood count (WBC). The y-axis represents the average effect of the WBC values listed along the x-axis. The pink sections are where the WBC pushes the prediction towards mortality while the blue sections are where the WBC pushes the prediction toward survival

SHAP force plots show the contribution of a single feature for one or several patients. Force plots for the patients in the test set for the features *temperature* and white blood count (*WBC*) are depicted in Figs. 6 and 7, respectively. The feature value is listed along the x-axis and is equivalent to the dot colour in Fig. 5. The y-axis shows the average feature contribution from patients with similar feature values.

All features are examined for all models. The aforementioned features highlight differences between the models and aspects to be aware of when developing and using ML models.

### SHAP individual force plots

**Fig. 8:**
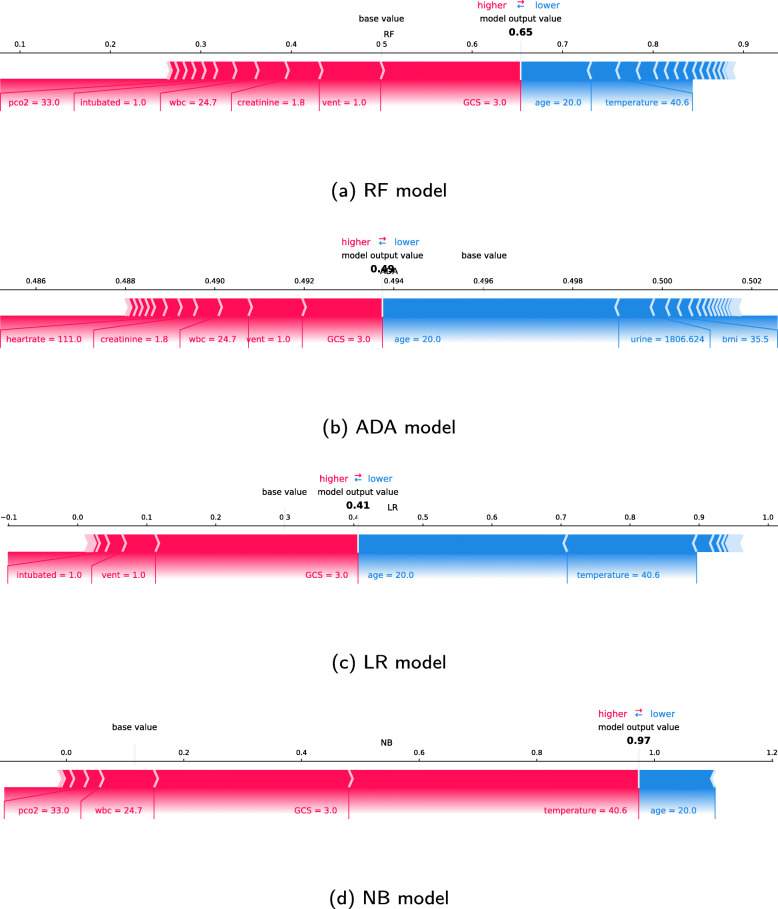
SHAP force plot for a selected patient (A). Features to the left of the model output value are pushing the prediction towards mortality while features to the right push the prediction towards survival

**Fig. 9:**
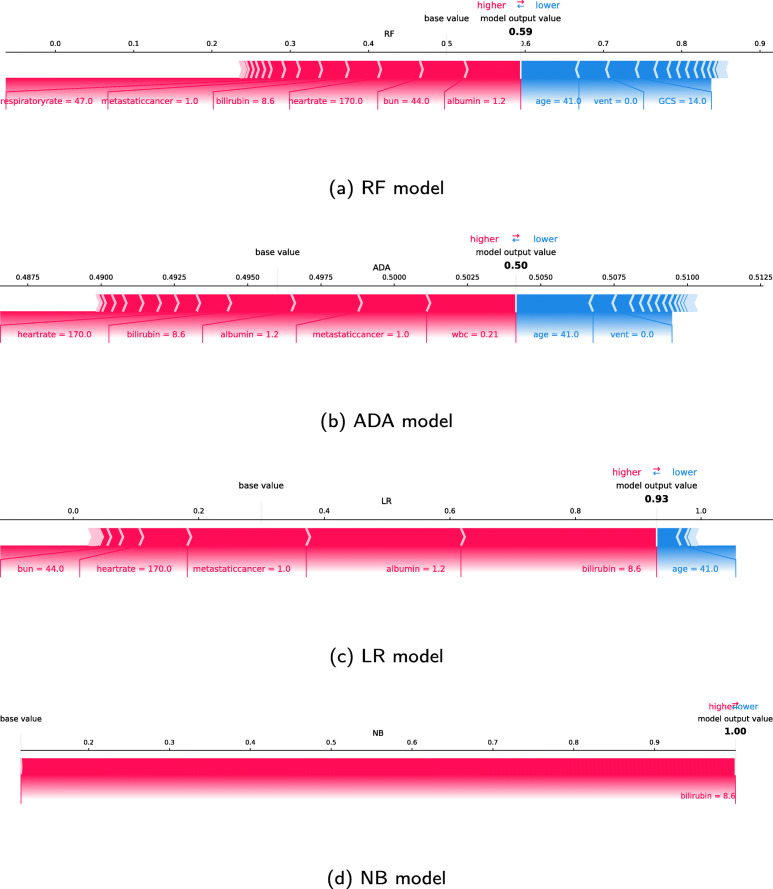
SHAP force plot for a selected patient (B). Features to the left of the model output value are pushing the prediction towards mortality while features to the right push the prediction towards survival

Figures 8 and 9 show the individual force plots for two patients (A and B) for the four different models; features the models consider relevant for the prediction of individual patients. The bold-faced number is the probability prediction (model output value), while the base value is the value that would be predicted if no inputs are given to the model. The blue features to the right of the prediction are the features pushing the prediction towards survival, while the pink features to the left push the prediction towards increased mortality. The length of the coloured segments helps visualise the size of the impact on the prediction. The longer the segment, the larger the impact. The length of the segments should not be compared between models.

Figure 8 shows the force plots for a patient (A) being alive at hospital discharge, which is predicted by the ADA, LR and APACHE IV model (prediction 0.39) if the risk threshold is 0.5. The RF and NB models predict that the patient dies. The models consider a low GCS score as the most, or second-most, influential factor concerning mortality. The RF, ADA and LR model also consider the fact that the patient is ventilated at the time of the worst respiratory rate as an important factor for mortality. The patient’s young age is the factor pushing the predictions most towards survival. While the models agree with the impact of several of the factors, they disagree with the influence of temperature. This patient has a high temperature of 40.6^∘^C, which is considered the second most prominent factor for survival by the LR and RF models.

The SHAP values for another patient (B) are shown in Fig. 9. With a risk threshold of 0.5, all ML models, as well as the APACHE IV model (prediction 0.54), predict that this patient dies in the hospital, which is also the case. Even though the models agree on the outcome, the SHAP values vary between the models. The age and the fact that the patient was not ventilated at the time of the worst respiratory rate are factors that push the prediction towards survival. After *age*, the LR model also considers the low white blood count as a factor pushing the prediction towards survival, followed by *vent* and *GCS*. The opposite is the case with the ADA model, where the low white blood count pushes the prediction towards mortality. A high bilirubin level is considered quite important by all models and is the only factor deciding the NB model outcome. The predictions given by the models are all above the risk threshold of 0.5.

## Discussion

### Area under the receiver operating curve

The overlapping confidence intervals show that the ML models have comparable discriminative abilities. The AUCs of these models are comparable to other published models [24, 25] and are therefore a good starting point for interpreting how these types of models work.

### Calibration curves

It is possible to calibrate the models during or after training. This is not done in this study in order to highlight the differences between models with similar AUCs more clearly. The calibration curve for the ADA model behaves differently from the others. The reason for this is that almost all predictions from the ADA model are probabilities between 0.48 and 0.51. Still, the model is good at discriminating between the two classes, and the AUC for the ADA model is higher than for the better calibrated APACHE IV.

The APACHE IV model is better calibrated than the other models but does not have the best discriminative abilities. The formula for calculating the APACHE IV score is not publicly available and is therefore not included in the rest of the comparisons.

### SHAP summary plot

Figure 4 show that chronic diseases are generally considered less important for the prediction than the other types of variables. Possible explanations for this are that chronic diseases are not affecting mortality to a large extent (independent of the model used), or due to a lack of samples since chronic diseases are rare. It is important to be aware that a feature that has a large impact on mortality in real life may be considered unimportant by a model. Some of the other binary variables are considered very important by many models, such as *vent*, whether the patient was intubated, and if the patient had elective surgery. The admission diagnosis does not seem to be considered very important by any of the models. The unit admit source is also not considered very important unless the source is the Operating room.

It is interesting to note how different models consider different features most important. This is particularly evident with the high importance given to *pCO2* by the NB model, which all other models consider significantly less important.

Considering Fig. 5, the NB summary plot is more right-heavy than the other models. Features contribute more towards increased mortality than increased chances of survival. The ADA summary plot looks different from the other models because of more discrete values rather than continuous, due to the mathematical fundamentals of the model. The ADA model summary plot looks less discrete if the number of decision trees used to build the model increases. However, more trees do not necessarily lead to improved performance.

### SHAP force plot

*Temperature* is listed among the top twelve features for all models as seen in Fig. 4. However, the impact of the temperature on the predictions differs between the models, as seen in Fig. 6. The force plots for the RF, ADA and LR models are divided into two distinct parts: A low temperature pushes the prediction towards increased mortality, while a high temperature is associated with higher chances of survival. The ‘switching point’ where the impact changes from negative to positive is around 36^∘^C. The linear behaviour of the LR model predicts increasing chances of survival with increasing temperature. The lower the worse, and the higher the better. The linearity gives a misleading relationship from a medical perspective, where too high temperatures also are deemed critical. The NB model reflects this nonlinear association. Values between about 35.5^∘^C and 38^∘^C are pushing the prediction slightly towards survival, while values outside this range push the prediction towards increased mortality. The impact increases with the distance from this range.

Figure 7 depicts the force plots for *WBC*. All models, except NB, have a ‘switching point’ for WBC between 10 and 15 1000/uL, where the impact of the WBC changes from contributing to survival to contributing to mortality. The WBC ‘switching point’ for the NB model is between 15 and 20 1000/uL. We can again observe the linear behaviour of the LR model, which does not correspond to medical theory. The RF and ADA models, and to a very small degree the NB model, do better reflect the negative effects of a low WBC.

The force plots for the other non-binary variables have similar behaviour as for *temperature* and *WBC*, and the force plots are in many cases divided into two distinct parts: Either low values push the prediction towards mortality, and higher values push it towards survival or vice versa. The LR model has a linear behaviour, while the shape of the RF, ADA, and NB plots varies depending on the feature. Even with different force plot shapes for the different models, the ‘switching points’ are often quite similar. This suggests that the models can provide insight into factors that have an impact on mortality.

### SHAP individual force plots

Considering the individual force plot in Fig. 8, the positive impact on survival corresponds to the force plots in Fig. 6. The NB model paints a different picture of patient A. The high temperature is here considered the most significant feature for increased mortality, which is in alignment with the common clinical sense, where high fever surely is not a good sign.

Considering the force plots for patient B in Fig. 9, the low *wbc* pushing the prediction towards survival for the LR model and towards mortality for the ADA model is consistent with the force plots in Fig. 7. The ML models agree on several of the factors pushing the prediction for patient B towards mortality. The ADA model gives a significantly lower probability than the other models, but when considering that the majority of the ADA model predictions lie in the interval [0.49-0.51], this prediction is among one of the strongest ones indicating a negative outcome.

The model outputs cannot be interpreted as probabilities because of the poor model calibrations, nor can they be compared between models. Individual force plots show which features the models consider relevant for the prediction of individual patients. However, even though we can visualise which features the models consider important for individual patients, the models are not developed for individuals, and the prediction will only reflect the average risk for patients with similar risk factors.

Combining the results, we can see that a better model in terms of AUC does not implicate a more accurate model in terms of medical theory and vice versa. A model can be good at one aspect and still fall short on others. An example is the NB model. The NB model has the worst AUC of the models developed, and as seen with patient B in Fig. 9 does the model only consider *bilirubin* when making a prediction. From a medical perspective, this is too little information to say something meaningful about the patient’s condition. Still, the NB model corresponds better to medical theory when it comes to the temperature and WBC as both too high and too low values push the prediction towards mortality.

### Limitations/future work

When developing ML models, there are nearly endless choices and trade-offs regarding variables, type of models, pre-processing techniques, and choice of hyper-parameters to name a few. Changing some of these factors may provide better models, but testing all combinations is not a feasible solution. For this work, we wanted to have a few, well-known robust models with similar performance to study and compare their transformation of inputs to prediction. For future work, we plan to also investigate deeper models with high AUCs.

This study shows differences between what the ML models consider important factors for mortality compared to medical theory. The reason behind these differences is a combination of several factors such as the underlying algorithm, choice of feature values, and pre-processing techniques. While the analysis of these aspects can provide valuable knowledge, this is outside the scope of this project.

The different performance metrics and visual representations of the data were limited to the ones considered most suitable. The SHAP Python library provides more visualisation possibilities, such as dependence plots to show global interpretability, which could be relevant for further analyses.

For a more complete comparison with already established models can APACHE II [26] or SAPS II or III [27–29] can be suitable alternatives since the formulas for calculating the scores are publicly available.

## Conclusions

The four different ML models developed in this study have similar discriminative abilities. However, further examination show that these models behave quite differently. While the calibration of the models illustrate that the predictions cannot be translated directly to probabilities, SHAP values show how the different models interpret different features.

Explainable ML models allow us to compare models differently than by using performance metrics like AUC alone. They also highlight important considerations for development and use that are otherwise frequently ignored. The SHAP plots enable visualisation of the contribution the individual features have on the prediction, and how the feature values push the prediction towards survival or death. Models using the same set of features can interpret the impact of these features differently and still have comparable performance, as seen in this study.

It is important to remember that the SHAP values do not imply causality; they merely give an insight into how the models consider various input, with respect to the output. While it is tempting to conclude that a variable is important for mortality since it has a high effect on the prediction, it is not necessarily the case. Furthermore, a seemingly good model does not necessarily correspond with a medically sound understanding. This study highlights this importance. No model is perfect, and acknowledging the limitations is an important part of development and implementation.

This study contributes to the growing area of explainable ML by exploring the differences between multiple ML models trained and tested on the same datasets. Black-box models are an enormous challenge within many fields where ML is both needed and utilised, and it is important to highlight challenges and limitation so that models can be used correctly and in the right setting. Understanding of how models work is also important for trust, which in healthcare ensure clinicians, patients and their families that the right decision is made.

## Data Availability

The dataset used is from the publicly available eICU database. The code for reproducing the work in this study will be published on GitHub (https://github.com/Explainable-Hospital-Mortality/individual-SHAP-comparison).

## Abbreviations

ADA: Adaptive Boost Classifier
AI: Airificial Intelligence
APACHE: Acute Physiology and Chronic Health Evaluation
AUC: Area under the receiver operating characteristic curve
BMI: Body Mass Index
BUN: Blood Urea Nitrogen
CGS: Glasgow Coma Scale
ICU: Intensive Care Unit
LR: Logistic Regression
ML: Machine Learning
NB: Naive Bayes
RF: Random Forest
SHAP: SHapley Additive exPlanations
WBC: White Blood Count

## Footnotes

1 Whether a patient is ventilated at the time of the worst respiratory rate.
